# AI-Powered Object Detection in Radiology: Current Models, Challenges, and Future Direction

**DOI:** 10.3390/jimaging11050141

**Published:** 2025-04-30

**Authors:** Abdussalam Elhanashi, Sergio Saponara, Qinghe Zheng, Nawal Almutairi, Yashbir Singh, Shiba Kuanar, Farzana Ali, Orhan Unal, Shahriar Faghani

**Affiliations:** 1 Department of Information Engineering, University of Pisa, 56122 Pisa, Italy; sergio.saponara@unipi.it; 2 School of Intelligence Engineering, Shandong Management University, Jinan 250100, China; zqh@sdmu.edu.cn; 3 Information Technology Department, College of Computer and Information Sciences, King Saud University, Riyadh 145111, Saudi Arabia; nawalmutairi@ksu.edu.sa; 4 Department of Radiology, Mayo Clinic, Rochester, MN 55905, USA; singh.yashbir@mayo.edu (Y.S.); kuanar.shiba@mayo.edu (S.K.); faghani.shahriar@mayo.edu (S.F.); 5 Department of Molecular and Medical Pharmacology, University of California, Los Angeles, Los Angeles, CA 90095, USA; fali@buffalo.edu; 6 Departments of Radiology, School of Medicine, Medical Physics University of Wisconsin-Madison, Public Health Madison, Madison, WI 53705, USA; unal@wisc.edu

**Keywords:** object detection, radiology, artificial intelligence, convolutional neural network, diagnostic accuracy

## Abstract

Artificial intelligence (AI)-based object detection in radiology can assist in clinical diagnosis and treatment planning. This article examines the AI-based object detection models currently used in many imaging modalities, including X-ray Magnetic Resonance Imaging (MRI), Computed Tomography (CT), and Ultrasound (US). The key models from the convolutional neural network (CNN) as well as the contemporary transformer and hybrid models are analyzed based on their ability to detect pathological features, such as tumors, lesions, and tissue abnormalities. In addition, this review offers a closer look at the strengths and weaknesses of these models in terms of accuracy, robustness, and speed in real clinical settings. The common issues related to these models, including limited data, annotation quality, and interpretability of AI decisions, are discussed in detail. Moreover, the need for strong applicable models across different populations and imaging modalities are addressed. The importance of privacy and ethics in general data use as well as safety and regulations for healthcare data are emphasized. The future potential of these models lies in their accessibility in low resource settings, usability in shared learning spaces while maintaining privacy, and improvement in diagnostic accuracy through multimodal learning. This review also highlights the importance of interdisciplinary collaboration among artificial intelligence researchers, radiologists, and policymakers. Such cooperation is essential to address current challenges and to fully realize the potential of AI-based object detection in radiology.

## 1. Introduction

Artificial intelligence (AI) in medical imaging has revolutionized healthcare through early diagnosis, improved prognostic accuracy, and streamlined workflows. Object detection in radiology refers to the identification and localization of regions of interest, such as tumors, lesions, or specific anatomical structures. This is key to assisting clinicians in decision-making and facilitating personalized treatment. Advances in imaging modalities, such as X-ray, Magnetic Resonance Imaging (MRI), and Computed Tomography (CT), have significantly improved image resolution and accessibility. However, analyzing these high dimensional and complex datasets is time consuming and prone to human error. AI systems help address these challenges by leveraging computational methods to detect patterns and abnormalities with greater precision and speed. By reducing inter-observer variability and improving reproducibility, AI plays a critical role in both clinical practice and research [1,2]. AI can assist radiologists by automatically identifying and highlighting suspicious regions in medical images, enhancing diagnostic workflows and reducing the risk of human error. However, AI-driven object detection goes beyond diagnostics. It enables automated monitoring of disease progression, preoperative planning, and real-time guidance during minimally invasive surgeries [3]. In oncology, for example, AI has been used to monitor tumor size and growth across multiple imaging sessions, enabling early interventions when needed. It also assists clinicians in selecting the best treatment plans through real-time image analysis. AI’s applications in large population screenings have been shown to reduce the burden on the healthcare system, especially in areas with limited medical expertise [2,4], and demonstrated that AI can outperform radiologists in breast cancer screening. Case studies of AI in radiology have proven this. For example, a deep learning system developed by Google Health has been shown to be better than human radiologists at detecting breast cancer in mammograms [2]. AI is also being applied to detect colorectal cancer using colonoscopy images where deep learning models analyze polyp images for early signs of malignancy, thus reducing human error in screening [5]. These case studies clearly indicate that AI can not only improve diagnostic accuracy but also serve as an early symptom detection technique, making it quite important for the efficacy of successful treatment. Despite its promise, the deployment of AI-based object detection in medical imaging remains hindered by several critical challenges. These include limitations in data quality, variability and inconsistency in annotation accuracy, and difficulties in achieving robust generalization across heterogeneous patient populations and diverse imaging modalities, all of which require further exploration [6]. For example, AI systems may perform perfectly on one dataset but fail to generalize when applied to images taken from different hospitals in different countries due to the diversity in imaging equipment, demographics, and disease prevalence. Similarly, ethics related to the privacy of data, algorithmic biases, and the interpretability of decisions made by AI systems need to be sorted out to ensure safe and equitable use in clinical practice. The ongoing debate concerns how AI algorithms should be governed and monitored to provide transparency in the decision-making process and prevent the perpetuation of biases inherited through historical data. While artificial intelligence continues to advance in the domain of image analysis, significant challenges remain in the development of algorithms that are both interpretable and explainable to clinicians. One key research area will be the development of “explainable AI” (XAI), which seeks to provide human-understandable reasons for AI system predictions. This is essential for fostering trust among health professionals and ensuring the responsible integration of AI into clinical practice [7]. Thus, any AI-driven radiology should not only be concerned with improvement in performance but also with other issues, such as fairness, accountability, and transparency in healthcare decision-making. The future of AI in medical imaging seems very bright as more data gets collected, algorithms become more sophisticated, and the role of AI in radiology continues to soar, enabling more personalized, efficient, and accurate delivery of healthcare. Advancements in AI object detection have also been able to present opportunities for early disease detection, real-time decision-making, and improved navigation for clinicians within the dynamics of modern healthcare. Convolutional neural networks (CNNs), a member of the deep learning family, extract subtle spatial features from imaging data, which in most cases are invisible to the human eye and make early detection via precision machines better. They thus help patients as they enable early identification of clinical indicators and facilitate timely referrals. Thus, a patient spends less time under diagnosis due to reduced operational processes by computerization via AI integration which could enable rapid radiology scans toward automated summarization to enhance clinical decision-making efficiency. Most importantly, a radiologist can devote more time to more complex cases resulting from automation, while some cases could allow for remote interpretation. As a result, patients spend less time waiting and receiving healthcare. This is because AI assistance integrated within the system facilitates more accurate diagnostic scans and summaries. Radiologists are better able to manage their workload because of the enhanced automation of simpler cases. Also, remote interpretation improves accessibility and efficiency for many healthcare professionals.

**Figure 1:**
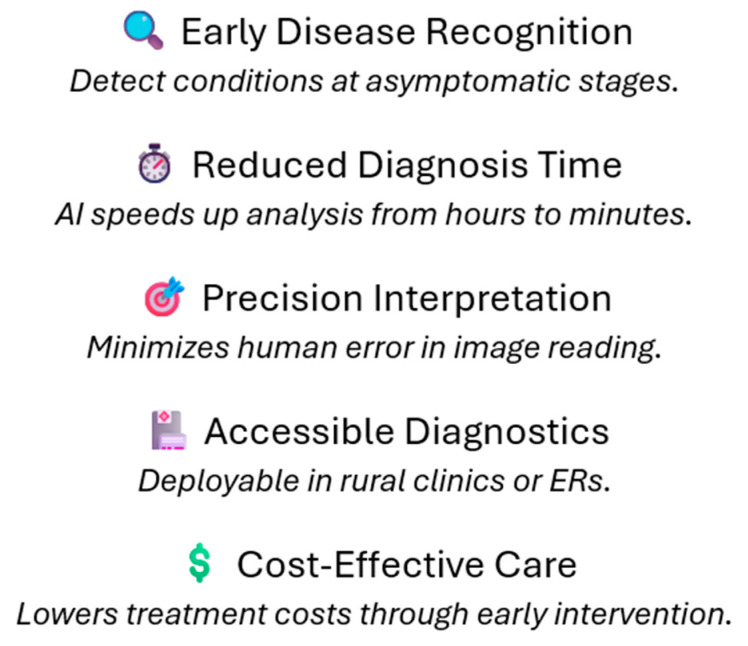
Overview of the enhancements in the medical imaging pipeline due to AI implementations: early detection of diseases through deep learning models (CNN), decreased time taken for diagnosis, higher accuracy of images, wider availability of diagnostics across the clinical field, as well as more rapid and cost-effective treatment. AI, on the other hand, also optimizes the radiology processes, improves the accuracy of interpretation, and widens accessibility to the diagnostic tool, particularly in rural regions.

In addition, there can also be more rural hospitals with reduced resources capturing an image via portable diagnostic tools and receiving reports from mobile applications. AI is beneficial in scaling hospitals by optimizing resources and workflows, thereby easing costs, and minimizing time wasted due to redundancies as illustrated in Figure 1.

The main contributions of this research are the following:

1. The review of the existing AI models in the area of object detection in radiology, with particular reference to their application in diagnostic imaging modalities such as X-rays, MRI, CT scans, and US within the specific frameworks of CNNs, transformers, and hybrid architecture, is provided in the paper itself.
2. Clinical applications of medical imaging object detection models are critically analyzed for potential benefits in terms of accuracy, robustness, and computational efficiency. Challenges concerning data scarcity, annotation quality, and the need for model generalization across population-based cohorts and different imaging protocols are discussed.
3. The contribution reviews critical privacy and ethical issues in the application of deep learning object detection in radiology, and these must be the very basis for secure and compliant solutions required in health AI. It also provides a rationale behind the need to deal with biases in AI algorithms, making them treat all patients equally by healthcare systems.
4. This review reveals new trends and future directions taken by AI research into radiology to assist collaboration among AI researchers, clinicians, and policymakers in bringing down the remaining barriers to the maximum benefit of AI techniques for improving diagnosis accuracy, patient outcomes, and modalities of medical imaging themselves.

After the introduction, Section 2 provides the overview of radiology modalities. Section 3 discusses the importance of image preprocessing. Section 4 presents current AI models for object detection. Section 5 explores data and annotation challenges. Section 6 examines the use of object detection in real-world applications for medical imaging. Section 7 highlights future directions and emerging trends. Finally, Section 8 presents the conclusions.

## 2. Overview of Radiology Modalities

**Figure 2:**
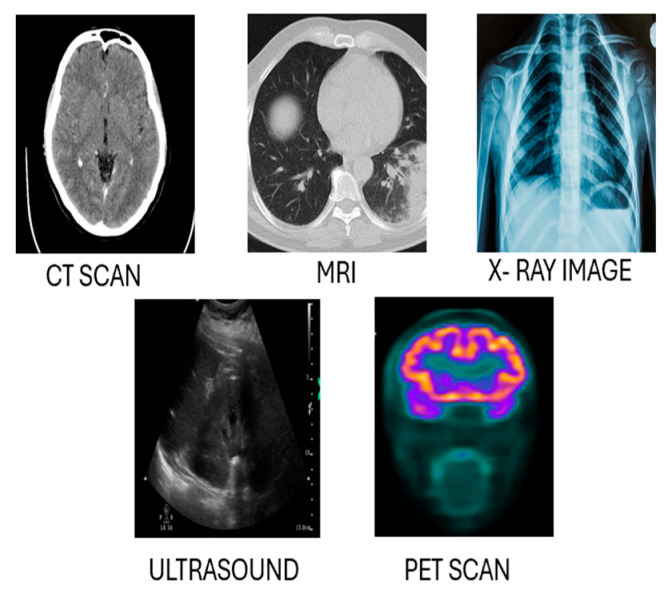
A comparison of different radiology techniques: X-ray imaging, PET (Positron Emission Tomography), CT (Computed Tomography), Ultrasound (US), and MRI (Magnetic Resonance Imaging).

Radiology represents the cornerstone of diagnostic procedures in modern medicine, allowing the investigation of specific structures inside the body and their related activities. The recent development of these techniques with the use of artificial intelligence allowed for more sophisticated imaging services in terms of quality and quantity. This part of the report describes, in general, the most relevant imaging modalities, concerning their characteristics, applications, and the development of AI-driven object detection. Comparative analysis and other systems are presented as proof of why AI is important in these techniques. Figure 2 illustrates examples of the diagnostic and therapeutic applications of CT, MRI, and other radiology modalities, alongside the information outlined above. All the presented modalities have underlying principles and their merits and limitations. Table 1 illustrates the key radiology modalities and their characteristics, highlighting their descriptions and common use cases. It provides an overview of how each modality, such as X-ray, CT, MRI, Ultrasound (US), and Positron Emission Tomography (PET), is utilized in diagnosing and monitoring various medical conditions.

Advancements in AI technologies, especially in deep learning models, have accelerated object detection of all types of medical images. Some key contributions of AI in these imaging modalities and case studies and benchmarks are described in Table 2. The use of artificial intelligence in different radiology modalities presents various opportunities and challenges, depending on the particularities of each modality. For example, in X-ray imaging, which is both inexpensive and quick in acquiring images, AI is useful in identifying pulmonary nodules and pneumonia. CheXNet [8] is a 121-layer convolutional neural network that achieves diagnostic performance comparable to that of radiologists in the detection of pneumonia, enabling non-invasive, image-based diagnosis with high accuracy. However, some inherent issues such as low resolution and the presence of organic tissue masks allude to the fact that there is a need for extra preprocessing methods and datasets, and more focus on the usage of CT imaging is required. Similarly, deep learning-based AI models demonstrate high efficiency in segmentation and quantitative analysis of CT images. For instance, Ref. [9] highlighted the effectiveness of 3D convolutional neural networks in enhancing lung nodule segmentation, thereby improving both diagnostic confidence and accuracy. However, the inherently high-radiation dose associated with CT imaging and the complexity of data processing required for extracting volumetric information remain significant challenges in clinical implementation.

AI has proven to be highly beneficial in advancing MRI techniques, enhancing its ability to provide exceptional soft tissue contrast. This makes MRI particularly valuable in diagnosing oncological and neurological conditions. Ref. [10] demonstrated the effectiveness of the U-Net architecture in achieving the best performance of the BraTS Challenge in brain tumor segmentation. However, challenges remain in reducing the acquisition time and mitigating motion-related artifacts in certain MR images. Real-time Ultrasound (US) imaging also relies heavily on AI although it can be problematic. Ref. [11] incorporated YOLO-based strategies for efficient lesion detection, though this can be counterproductive as US imaging heavily depends on the operator, leading to variability in image acquisition and quality. Relying on that strategy brings forth the problem of excessive speckle noise which requires robust noise and consistency improvement frameworks to solve. PET imaging has advanced with the introduction of AI, especially in detecting cancer at its initial stages and mapping brain activity. Ref. [12] integrated CNNs with recurrent neural networks (RNNs) to sequence PET images, enabling improved early diagnosis of Alzheimer’s disease. A critical limitation to the broader adoption of this technology is its dependence on radioactive tracers. Developing alternative methods that achieve comparable diagnostic performance without tracers could significantly enhance its clinical utility and accessibility. Moreover, these imaging modalities face broader challenges, including the scarcity of annotated datasets for rare pathologies and the need for robust, generalizable AI models capable of performing reliably across diverse patient populations and imaging platforms. A challenge in the clinical adoption of deep learning lies in the limited interpretability of complex neural networks and the regulatory constraints imposed on core healthcare systems. While certain AI models may be rapidly deployed for specific tasks, their integration into clinician-assistive workflows requires extensive validation and explainability. Federated learning offers a promising framework for collaborative model development across institutions, enabling data privacy preservation while enhancing generalizability. Explainable AI (XAI) initiatives that aim to bridge the gap between models and clinician trust provide transparent, actionable insights into model predictions. Furthermore, the growing demand for computational resources highlights the need for designing energy-efficient AI models to reduce environmental and infrastructural burdens. Finally, large-scale integration of electronic medical records across platforms may significantly enhance diagnostic workflows. Leveraging the complementary strengths of diverse imaging modalities and equipment, a more comprehensive and accurate patient-centered diagnostic approach, leads to improved health outcomes.

## 3. Importance of Imaging Preprocessing

**Figure 3:**
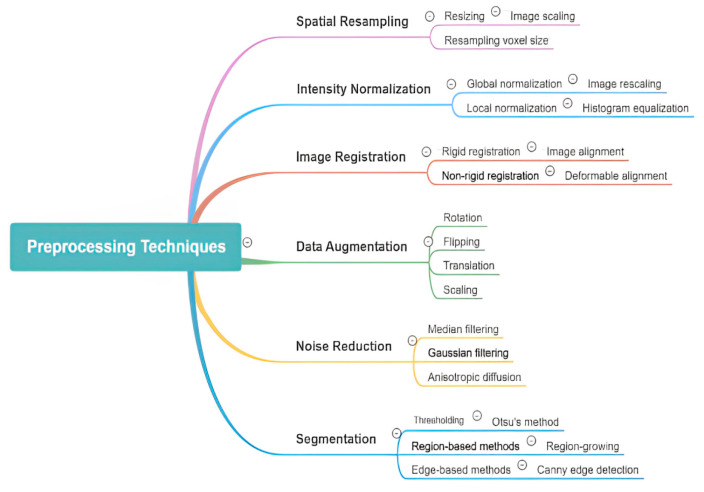
Overview of various image preprocessing techniques used in AI-powered object detection in radiology. The diagram categorizes preprocessing methods into key areas, including spatial resampling, intensity normalization, image registration, data augmentation, noise reduction, and segmentation. Each category further details specific techniques such as resizing, histogram equalization, deformable alignment, flipping, Gaussian filtering, and region-based segmentation, highlighting their roles in enhancing image quality and improving model performance.

Preprocessing procedures applied prior to algorithmic analysis are important for enhancing the performance of AI models in medical image object detection. These steps improve both the efficiency and accuracy of deep learning models by addressing common challenges, such as image noise and artifacts and protocol variability. Due to the complex and heterogeneous nature of medical imaging, robust preprocessing techniques are essential to effectively analyze and interpret medical images. Noise reduction is a primary goal, with techniques like Gaussian filtering, median filtering, and wavelet transformations utilized to preserve relevant anatomical structures. Additionally, enhancement methods such as adaptive contrast and histogram equalization can improve image clarity, enabling more precise differentiation between normal and abnormal features. AI models also ensure the regulation of differences in intensity values and resolution to guarantee consistency across datasets, through techniques such as min-max and z-score normalization. Edge detection, inpainting, and region-of-interest (ROI) extraction are some of the artifact removal techniques that help to reduce the AI algorithms’ misinterpretations that are caused by erroneous motion, implants, or scans. In addition, segmentation and region extraction techniques such as thresholding and morphological techniques improve the focus of AI models on relevant biological structures, which in turn increases the efficiency of computation. Deep learning AI models are able to more easily spot tiny patches of disease because of resolution improvement techniques like interpolation and super-resolution. The medical images are geometrically transformed by rotating, scaling, and flipping to make them position invariant. Some of the data augmentation procedures applied include flipping, rotation, cropping, and elastic deformation of the original images. These techniques help in creating larger datasets for training of the model and combatting the overfitting problem. Image preprocessing is very important, but it is difficult due to the differences in the imaging techniques used, too much processing power used, the risk of missing out important clinical details, and the need to have automated systems that can change themselves as needed. More work should be made in creating AI-propelled adaptive image preprocessing systems that use deep learning techniques like GANs for denoising and super-resolution, making the standards for post-processing uniform across the board and using knowledge from specific fields to enhance important patient findings and features [13]. Effective data preprocessing plays a crucial role in the efficacy of a diagnostic model, as it directly impacts clinical outcomes. Therefore, it is essential for AI-based object detection approaches in medical imaging. Various image preprocessing methods are required for AI-based object detection in orthopedic imaging as shown in Figure 3. They include spatial resampling, intensity normalization, image registration, data augmentation, noise reduction, and segmentation. Among these, some tactics entail resizing, histogram equalization, deformable alignment, flipping, Gaussian filtering, and region growing. Through enhancing image clarity, noise reduction, and highlighting zones in the foreground, these methods help with easy interpretation and analysis of AI-based models.

## 4. Current AI Models for Object Detection

**Figure 4:**
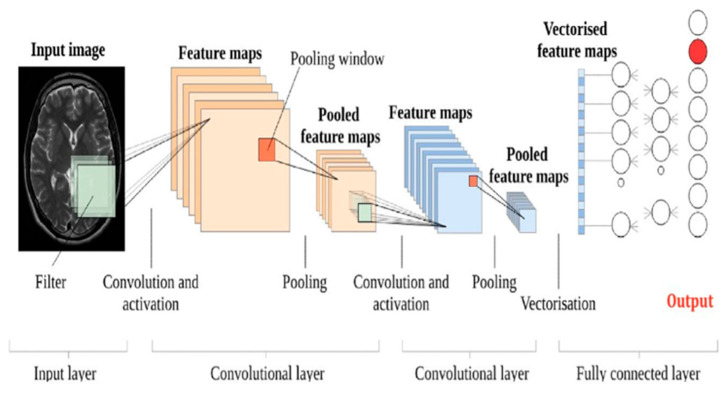
A sample of a CNN architecture designed for a brain MR image. The diagram illustrates the input layer (medical image), followed by convolutional layers, pooling layers, fully connected layers, and the final output layer [14].

AI use in radiology has seen a surge in the past few years, specifically in object detection tasks. In radiology, object detection aims to find specific objects such as tumors, lesions, or other organs of interest in medical scans, including X-rays, MRIs, and CTs. One of these innovations is the CNN-based approach of segmenting MRI images. It is initiated by the input layer taking an MRI scan. This specific step is performed after several convolutional layers, where features are taken from the images of MRIs based on convolutions of an input by a matrix of weights. These types of features possess a sense of limit of collaboration in a high sense of active sense, which is one of the most active shapes of features. Enhancing collaboration contributes to improving techniques such as MRI, which support better textural control and image comprehension. After this stage, pooling layers are introduced which decrease the spatial dimensions of the feature maps in order to maximize reductions in computation. Pooling has set a balance between ordinary pooling and average pooling while still retaining important information. The extracted features are now passed through completely connected layers in order to understand and create images with even higher resolutions. Usually, these layers will have the parameterization of instance encodings for a classifier or a regressor based on the segmentation task. The goal is to be able to construct an adequate CNN for medical image segmentation, that is, the network will produce a segmented image where the output gets encoded with a specific label that details the region of interest on the MRI as some type of tissue or many types of malformations. Figure 4 illustrates a sample of CNN architecture designed for brain image.

The image processing procedures, like many others, have highly benefited from the use of cutting-edge AI systems, including deep learning networks, ensemble models, and AI systems based on LLMs. Nonetheless, the interpretability of the model, its scalability, and its robustness to varying conditions in radiology remain challenges. Agentic object detection in radiology exploits the best of AI algorithms to identify, localize, and recognize, in an autonomous manner, the abnormalities that may occur in medical images, be they tumors, fractures, or lesions. Agentic systems ensure feedback learning and active adaptability, improving their accuracy on the fly in comparison with traditional methods. Following this trend, only the introduction of deep learning and reinforcement learning helps these systems to improve precision in diagnosis, cut potential radiologist workloads in half, and drive timely decision-making processes. Potential use of this technology lies in the improvement of diagnostics early on and personalizing treatment planning in radiology. We examined the existing artificial intelligence models for radiology object detection through ten different heterogeneous case studies based on LLMs, ensemble methods, and deep learning. Additionally, we discuss the advantages and limitations of these methods, evaluate their comparative performance, and outline potential future directions for AI-supported object detection in medicine. In Table 3, the gathered data are consolidated and compared according to the advantages and disadvantages of the models mentioned.

Various AI-based object detection models applied in radiology, including their technical advantages and limitations, are looked at in the table below. As explained in the study, YOLOv4 [15], a more advanced algorithm, is able to work on detecting and diagnosing these tumors in CT images with large tumors, a large field of view, and LAMPUS hardware that is extremely difficult to manage where speed is an issue within clinical settings. However, geometric models encounter significant limitations in accurately localizing small tumors and differentiating them from image noise, especially when operating on low-resolution or degraded image data. Recent advances have led to the development of more robust object detection models, such as Faster R-CNN, which has significantly enhanced the practical applicability of this technology. The integration of RPNs within Faster R-CNN contributes to improved localization accuracy; after domain-specific fine-tuning, the majority of region of interest (ROI) predictions reliably correspond to relevant targets, although the precision of ROI detection remains dependent on the characteristics of the training dataset. Additionally, the model’s object classification component leverages a deep convolutional neural network (CNN) architecture capable of detecting clinically significant patterns, such as breast cancer in mammographic images [16]. However, further performance improvements through model stacking or boosting are constrained by the substantial data requirements for effective training and preprocessing. As a result, the advancement of detection performance increasingly relies on leveraging architecture-specific innovations rather than ensemble strategies. RetinaNet, for example, represents a state-of-the-art convolutional framework that balances speed and accuracy in object detection tasks through its use of focal loss to address class imbalance [17]. It addresses class imbalance with focal loss; the focal loss goes one step further, requiring huge backprop of the hard example weight focal loss class imbalance by using the iteration update of the Amplified Softmax output for classification of the L2 regularization with iterative learning. Only after that, whatever cascade is produced should be utilized, whether it is the best choice or a good one. Higher order kernels recognize an even more subtle form of geometry or appearance, holding promise for context integration across areas, given the moving spatial scale. Fully connected layers apply classification or regression and resolve above spatial relations in a higher-dimensional distribution of extracted features; a complete linear operation over the transformed features is provided by this. Injecting semantics and converting them into something as different from a fully connected network as the architecture allows are the domain properties and boundary conditions of the last module. Broadly stated, connectivity conveys the image pose, perceptual experiences, and depth of features amassed within the dense layer. However, this strength becomes a weakness, perhaps, in circumstances where the quality or resolution of X-ray images is subpar. Meanwhile, pixel-level segmentation of tumors from a brain MRI is conducted extremely well with the use of U-net architecture, which has a highly precise and accurate output on brain tumor detection and localization [18]. Its primary weakness is found in the overfitting that happens with smaller datasets as well as greater noise in images that have not been trained. EfficientDet, on the other hand, is focused on performance and speed, which is absolutely necessary when targeting minimal computational power for identifying cardiac abnormalities [19]. In addition to that, recent work has explored conditional generative adversarial networks C-DCNN to synthesize and augment brain tumor datasets. These studies found that synthetic data generated by C-DCNN significantly enhanced CNN classification accuracy by balancing class distributions and reducing overfitting, thus offering a promising direction for rare tumor subtype detection [20]. The drawback is the detection of internal structures and organs in the presence of external complex image backgrounds. The addition of LLMs to Vision Transformers has great potential in enabling multi-organ detection using a mechanism of transformers to conduct multiple tasks in a single architecture [21]. The real-time deployment of these algorithms is constrained by their substantial computational demands, including high processing power and large data requirements.

Tracking functions over time, such as the monitoring of disease evolution, could be important in the context of analyzing real-time data using Fast R-CNN combined with LSTMs [22]. However, large datasets are still a huge problem. The sequential information within the data has temporal dimensions, leading to longer operational latency, and in turn makes monitoring much harder. Finally, with multi-scale context capturing convolutional DeepLabV3+, the most accurate results for the detection of brain lesions in MRIs, where the variability of lesion increases the most, were obtained [23]. The model shows some merits, although it is too complex and expensive and requires additional calibrations. As a result, it is very hard for this model to be applicable in a real-time clinical environment. At the same time, serious problems about performance measurement in genuine clinical conditions are stated. Ref. [24], on the other hand, stepped in with the utilization of ensemble CNNs to search for colon polyps in colonoscopy images. Radiology tasks stand to benefit the most from CNNs due to ensemble methods, a means of combination that tends to be the most promising integration of several individual models to boost a single-task performance. By leveraging an ensemble of diverse model architectures, this approach allows for exposure to a broader range of data patterns and feature representations, thereby enhancing the model’s generalization capabilities and improving the overall detection performance. A key objective of the framework is to enhance generalization, especially in the context of complex colonoscopy images, which exhibit variability in image illumination and polyp appearance. This approach involves significant computational demands, as it requires both training and inference through multiple networks. Careful selection and integration of individual architectures avoid overfitting and minimize unnecessary complexity introduced by ensemble models. Consequently, the method could cause a lag in real-time processing due to extra computational overhead. Nevertheless, despite these problems, the improved performance observed by exploiting the ensemble CNN not only enhances colon polyp detection but also enhances the early-stage screening of colorectal cancer.

In their study on liver lesion detection in CT images using Mask R-CNN, Ref. [25] emphasizes the critical role of enhancing segmentation accuracy. Mask R-CNN, an extension of Faster R-CNN, integrates pixel-wise segmentation with object detection, making it especially suitable for tasks requiring precise lesion boundary delineation. A key strength of Mask R-CNN lies in its ability to produce high-resolution segmentation masks, which are critical for precise lesion detection and improved diagnostic accuracy. Accurate mask generation plays an important role in facilitating lesion classification and ensuring that the areas delineated regions are clinically meaningful. The Mask R-CNN is a popular and well-established model; however, it struggles with small or low-contrast artifacts, which are very common in MRIs. Due to the poor similarity and size scaling of the lesions, the model rarely generates accurate segmentation masks in such cases. Moreover, Mask R-CNN’s low interpretability paves the road for its high complexity, making it computationally expensive and reliant on significant overpower requirements such as the real trouble with its application in real-time scenarios and situations that are already resource-challenged. It is, however, definitely a major step toward state-of-the-moment liver lesion segmentation and hence the corresponding prospect of malignant liver tumor diagnostic indication and surveillance in the radiology domain.

## 5. Data and Annotation Challenges

Medical image annotation for AI model development is a complex process with numerous challenges affecting the efficiency, accuracy, and scalability of dataset preparation. Annotation of medical images is indeed a significant milestone, though exceptionally difficult in ensuring the maximum potential of developing AI models in healthcare. Two of the most widely used annotation tools, 3D Slicer and ITK-SNAP, address the challenges of medical image segmentation from distinct perspectives. The 3D Slicer is a comprehensive, open-source platform that supports multimodal imaging and provides advanced tools for 3D volumetric segmentation, making it suitable for complex tasks such as tumor annotation. However, its steep learning curve and high demand for expert user input can hinder efficiency and scalability. In contrast, ITK-SNAP emphasizes semi-automated segmentation, substantially reducing manual workload in anatomical delineation and offering a more user-friendly interface. However, it lacks robust support for large dataset processing and provides minimal collaborative features, limiting its applicability in high-throughput or collaborative annotation workflows. In addition to the primary challenges of data privacy and heterogeneous data formats faced by both tools, there is a need for standardized, secure workflows to enable reliable and reproducible AI model development. Ten key challenges have been identified, along with corresponding solutions to enhance annotation practices for medical image datasets. These challenges range from data preparation and annotator expertise to privacy concerns and scalability. The comprehensive examination of these studies provides critical insights into framing the data preparation pipeline and annotation processes, which are the solid basics of support for the development of reliable, and precise machine learning models, as is depicted in Table 4.

The above-mentioned studies address the challenges and opportunities in preparing and annotating medical datasets for machine learning. Ref. [26] highlights the unnecessary time spent on data preparation, such as cleaning and formatting, and suggests that the design of efficient preprocessing pipelines can speed up the dataset availability phase and increase consistency. Ref. [27] points out that annotators must have expertise in the use of the annotation tool and knowledge in the healthcare domain, which requires them to be trained and receive support from clinical staff for proper labeling, which enhances the data quality. Ref. [28] deals with the quality of the labels and points out the need for labeling consistency, recommendation standardized protocols, and quality control for labeling and to avoid discrepancies in the model training. Ref. [29] states the problem of an inadequate number of medical images and offers the establishment of data-sharing agreements combined with federated learning to increase images while respecting privacy regulations. Ref. [30] explores data bias which is especially prevalent in some demographic groups. Ref. [31] addresses the mitigation of black-box issues and stresses on proper anonymization and administrative exchangeable de-identification of data to maintain patient data confidentiality. Ref. [32] assesses the functioning of the annotation tool and argues for compatible and multifunctional sophisticated tools that enhance the automation of the annotation while minimizing oversight. Ref. [33] points out the necessity of continuous manual quality control, suggesting multi-tiered scrutiny and machine-assisted techniques toward efficient high quality annotation. Ref. [34] faces the problems of generalizing the process of annotation for big databases. Or rather, they propose the idea of crowdsourcing substantial amounts of annotating data with the supported structure of the annotation advanced computer systems. Ref. [35] focuses on the problem of integrating annotated datasets more seamlessly into the working environment of medical staff to encourage the use of AI tools in clinical workflows. They claim that there are several ways to enhance productivity and accuracy, as well as to increase the number of medically annotated images. This also emphasizes that data preparation, the quality of the data annotation, data privacy, and the integration of data into clinical practice are all interrelated.

## 6. Real-World Implementation

The use of AI-powered object detection in medical biological imaging significantly enhances the accuracy and speed of diagnosis. However, its implementation in real-life settings face several barriers, including, but not limited to, computational, ethical, integration, performance, and economic challenges. Key challenges to real-world deployment of AI in clinical settings include image variability, limited computational resources, dataset bias, and the need for clinician acceptance. Table 5 provides an overview of the major gaps in the application of artificial intelligence in radiology and suggests remedies that can maximize performance and mitigate bias, compliance, and adoption. AI-based radiology deals with a myriad of issues pertaining to the computational, ethical, integration, performance, and economic realms that are each resolved by the implementation of specific trade-offs. Problems in computation, which include generalization of the model and constraints in resources, occur due to variation in image quality, resolution, and acquisition parameters among different imaging modalities [36]. Models that are built on high-resolution MR images turn out to be inadequate even when used with low-resolution MRI [37] requiring architectures and domain adaptation approaches [38], but they have the adverse effect of adding more complexity and computational demands. Also, on-the-fly processing in environments with reduced resources [39] is an obstacle with AI-enhanced US systems lagging [40]. Improvement in these cases comes through techniques that, like quantization and pruning [41], fasten the process, but they have side effects like accuracy and robustness decrease.

Imbalanced datasets generally lead to a variation in diagnostic accuracy between populations, as pointed out by [42]. For instance, AI models for breast cancer detection have been reported to be less accurate for African patients, as reported in [43]. Researchers emphasize the requirement of utilizing diverse datasets and fairness-aware training techniques to mitigate these challenges, as discussed in [44]. The requirement for AI models to be both fair and diverse is influenced by privacy and regulatory concerns. The ongoing ambiguity and liability in cases where AI errors lead to incorrect diagnosis [45] highlight the risks, such as AI misclassification in stroke detection affecting treatment decisions [46]. Establishing principles for the use of AI with standard protocols for the diagnosis of errors [47] is inevitable, but it is a goal that requires worldwide concord. The adoption of AI is also hampered by challenges in its integration into health systems, such as interoperability and clinician acceptance. AI tools are often incompatible with existing healthcare systems, such as Picture Archiving and Communication Systems (PACSs) and Electronic Health Records (EHRs) [48], leading to system failures [49]. By following standards like DICOM and HL7 FHIR and collaborating with providers, organizations may be able to ease integration, but this approach requires many resources. The anguish of patients and particularly clinicians caused by the black-box AI’s approach [50] is the main issue, while lung nodule detection AI [51] only adds fuel to the fire. In these situation, expectations of explainability and a user-friendly format [52] are the main needs; machine learning model (ML)-based strategies get complex with acceptance and interpretation. One permutation of performance challenges are variations in the imaging conditions [53] endorsing abuse examples in which motion artifacts generate major false positive rates [54] although in data augmentation and preprocessing it is difficult to replicate real-world variability [55]. Rare case handling is also an issue due to the lack of detection from missing classes in training [56], underlining the inviability of rare subtype detection in glioblastoma [57] but also synthetic data generation [58] or transfer learning, while families, due to generative data, are not quite so verisimilar. Hurdles such as high expenditure and training the workforce represent economic deterrents to adoption, with the implementation of AI being essentially unaffordable for a tertiary hospital presented an upgrade of USD 1 million [59], resulting in system [60]. Public–private partnerships [61] can provide valuable support for AI integration but require ongoing maintenance. A lack of familiarity among non-expert users [62] prolongs the learning for radiologists, who often require extended training periods [63], underscoring the need for comprehensive training programs into medical school curriculum [64]. Finally, successful integration of AI in radiology requires navigating trade-offs between performance, interpretability, ethical concerns, and economic feasibility.

## 7. Future Direction and Emerging Trends

A future direction tends to be very promising at the blending of AI with AR and VR. These technologies can enhance medical image 3D visualization, improving the interpretation of data and consequently allowing clinicians to make a more accurate diagnosis. For instance, AR could overlay the surgical instruments as well as the needed remedies of proficiency over the patient during surgery for real time guidance. Medical staff are also trained through VR by simulating different kinds of medical emergencies, which help them visualize the scenario and prepare them for the actual experience. This melding can significantly increase the accuracy and efficiency in surgical procedures. Application in applied medical sciences has shown promising benefits in AI, VR, and AR due to the increase in predictive and diagnostic performance. Educational outcomes also increase by improving quality. AR technologies in healthcare can revolutionize surgical planning and assist by projecting 3D anatomy models on a patient for safer and much more precise techniques. Medical training with the help of VR becomes more engaging as students are given hands-on supervision, ensuring they never forget the procedures [65,66,67].

### 7.1. Wearable Devices and Real-Time Monitoring

Wearable devices are capable of providing continuous monitoring for patients, including real-time diagnostics and alerts. For instance, wearable ECG monitors can detect abnormal heartbeats and send alerts to healthcare professionals in real time. This could greatly improve triage and treatment, allowing people to receive care sooner. This is achieved because these devices allow gaps in observation to be easily controlled, aided by the fact that these gadgets will have built-in AI in the future. AI-powered wearables are revolutionizing healthcare systems around the world by enabling real-time continuous monitoring various vital parameters and providing alerts to users and caregivers: Wired and wireless ECG machines have transformed cardiac care beyond the confines of a hospital by enabling the identification and monitoring of abnormal heart rhythms, sensing irregularities, and sending alerts to caregivers for timely intervention. Continuous monitoring through wearables closes gaps in observation can lead to improved outcomes. The AI technology integration with wearable health gadgets is predicted to advance toward more complex observing and diagnosing capabilities in the future [68,69,70,71].

### 7.2. Regulator and Ethical Considerations

With AI systems processing confidential medical data, safeguarding data privacy and security is critical. Regulations must be put in place to protect patient data and ensure compliance with privacy legislation. AI algorithms should learn without any biases to provide fair and equitable healthcare. However, biased training data can lead to unequal healthcare outcomes. To address this issue, researchers and developers of the future will need to work toward creating transparent and unbiased AI systems that clinicians and patients can trust. With AI algorithms becoming increasingly widespread in existing healthcare systems, regulatory frameworks are critically important to ensure that sensitive medical data are handled appropriately and in adherence to the privacy laws outlined by the World Health Organization. Ensuring that AI functions fairly and equitably is imperative. Biases can and will still occur, however, if the training data are not properly representative of the actual population, disparities will occur. Future work should ensure that AI systems are transparent and as free of bias as possible, so they can be trusted by clinicians and patients alike [72,73,74,75].

### 7.3. Advances in Deep Learning Algorithms

More advanced neural network transformers currently being developed are improving the precision of object detection in radiology. These sophisticated algorithms enhance diagnostic accuracy by analyzing complex patterns in medical images. Additionally, unsupervised learning methods are being explored to reduce the need for annotated data, thereby simplifying the training of artificial intelligence models. Future advancements in deep learning are likely to improve the efficiency and accuracy of AI-driven object detection. The development of sophisticated neural networks including transformers has greatly improved medical image object detection accuracy. Advanced algorithms can identify complex patterns in medical images and lead to more accurate diagnoses. Unsupervised learning methods are being investigated to decrease the need for annotated data, therefore simplifying AI model training. Future developments in deep learning are projected to enhance the accuracy and efficiency of object detection run by artificial intelligence [76,77,78,79].

### 7.4. Explainable AI (XAI)

XAI is, therefore, vital for building trust in AI systems. To make informed decisions, clinicians need to know how and why an AI prediction has been made. This paper contributes XAI techniques to improve the transparency and understandability of AI decisions, making them more acceptable in clinical practice. Furthermore, the transparency of the AI can also facilitate the identification and correction of biases in the algorithms used. It is further predicted that future research will be concerned with the development of more complex XAI techniques in order to obtain more detailed information about the processes of decision-making in the AI systems. XAI is important for increasing the transparency and trust of AI systems, especially in healthcare. XAI is important because it enables the clinicians to understand which predictions the AI is making and thus what decision they should make. The XAI techniques seek to make the decisions of the AI systems transparent and understandable with the aim of recommending them for clinical practice. This transparency in the AI may also help in identifying and correcting the biases in the algorithms used in the healthcare system. The future research is likely to focus on how to develop more sophisticated XAI techniques that can give a deeper understanding of how AI makes decisions [80,81,82,83].

### 7.5. Cloud Computing and Big Data Analytics

Cloud computing has several advantages for radiology by providing scalable resources for processing and data storage. Cloud platforms can handle large scale data analysis, enabling the efficient processing of vast amounts of medical images. This computational power is essential for developing and deploying AI models. Additionally, big data analytics presents numerous opportunities to generate new insights from medical images, ultimately improving diagnosis and treatment planning. The integration of cloud computing and big data analytics is expected to play an increasingly critical role in advancing AI-powered solutions in radiology. Cloud computing infrastructure provides scalable computational and storage resources essential for managing the vast volumes of imaging data radiology, therefore enabling more efficient data processing and AI model development and deployment. Big data analytics enables the extraction of clinically relevant patterns and insights from large imaging datasets, improving diagnostics and treatment planning. Together, these technologies can enhance the performance and scalability of AI powered applications in radiology [84,85,86].

### 7.6. Integrating AI-Powered Object Detection into Clinical Workflows

Effective deployment of AI-powered object detection requires seamless integration into the clinical workflows. This necessitates educating healthcare practitioners on the effective and practical use of AI tools. Equally important is the development of user-friendly interoperable systems that seamlessly integrate into daily clinical workflows. Addressing these barriers is essential for the sustainable and widespread usage of AI in radiology. The next phase will likely involve the development of comprehensive training programs and easy-to-use intuitive interfaces, ensuring widespread implementation of AI in clinical settings. Deploying AI-powered object detection during clinical workflows is vital to its efficiency, however careful consideration and execution are required. To achieve this, it will be imperative to train healthcare professionals on AI-driven applications and make them usable within clinical workflows in a manner that is second nature. User-friendly systems also play into accommodating human factors necessary for widespread adoption. Addressing these challenges for the mainstream integration of artificial intelligence tools in radiology will likely require advancements in both technology and human factors. Moving forward we will likely be focused toward nimble training programs implemented at scale along with easy-to-use interfaces as enablers helping further propagate the presence of AI technologies within clinical settings [87,88,89].

## 8. Conclusions

The use of AI in medical imaging has transformed the landscape of healthcare, particularly in diagnosis, treatment planning, and patient care. Deep learning models such as CNNs and transformers have been capable of demonstrating outstanding accuracy and efficiency in the identification of pathological features on various imaging modalities, including X-ray, MRI, CT, and US. These advancements not only provided more precise diagnoses but also aided in real-time decision-making and tailored treatment plans, hence enhanced patient outcomes. However, despite these advances, there are certain challenges that still exist. Sparsity of data, annotation quality, and the need for robust, generalizable models across populations and imaging protocols are some of the challenges that have to be addressed in order to make AI acceptable in clinical practice on a large scale. Ethical and privacy concerns in patient data use and AI-driven decision-making are also significant to address in order to make AI technology safe and fairly deployed in healthcare. Future studies should focus on creating lightweight AI models for low-resource settings, using federated learning to support privacy-enhancing use cases and using multimodal data to improve diagnosis. Successful integration of AI in radiology will require cross-disciplinary cooperation between AI researchers, clinicians, and policymakers to address existing challenges and develop innovation.

## Figures and Tables

**Table 1:** Radiology modalities and their characteristics.

Modality	Description	Common Use Cases
X-ray Imaging	Uses electromagnetic radiation to obtain pictures of structures in the body, such as bones and lungs.	1. Fracture diagnosis. 2. Diagnosis of chest abnormalities (e.g., pneumonia, tuberculosis). 3. Disease surveillance.
Computed Tomography (CT)	Uses X-rays and a computer to produce cross-sectional views of the body’s anatomy.	1. Tumor or cancer detection. 2. Vasculature imaging. 3. Trauma injuries imaging.
Magnetic Resonance Imaging (MRI)	Uses magnetic fields and radio waves to produce detailed images of soft tissues.	1. Disorders of the brain and spinal cord imaging. 2. Diagnosis of joint injuries. 3. Soft tissue tumor detection.
Ultrasound	Utilizes high-pitched sound waves to provide real-time images of organ movement and blood flow.	1. Fetal development observation. 2. Diagnosis of abdominal or pelvic conditions. 3. Examination of blood flow in arteries and veins.
Positron Emission Tomography (PET)	Involves the injection of a radioactive tracer to visualize metabolic activity in tissues.	1. Identification of cancer and monitoring treatment response. 2. Evaluation of brain disorders (e.g., Alzheimer’s). 3. Evaluation of heart function.
X-ray Imaging	Uses electromagnetic radiation to obtain pictures of structures in the body, such as bones and lungs.	1. Fracture diagnosis. 2. Diagnosis of chest abnormalities (e.g., pneumonia, tuberculosis). 3. Disease surveillance.

**Table 2:** AI techniques and performance metrics across radiology modalities.

Modality	AI Techniques Used	Key Contributions	References	Performance Metrics
X-ray	CNNs, Transfer Learning	Automated detection of lung diseases (e.g., COVID-19, tuberculosis)	[8]	Sensitivity: 90%, Specificity: 87%
CT	3D CNNs, Attention Mechanisms	Tumor segmentation and volume measurement	[9]	Coefficient: 0.85
MRI	GANs, U-Nets	Tumor detection, anomaly localization	[10]	Accuracy: 93%, Recall: 91%
Ultrasound	YOLO, Region-based CNNs	Real-time detection of lesions, fetal abnormalities	[11]	Precision: 88%, F1 Score: 0.89
PET	Hybrid CNN-RNN Models	Early cancer detection, brain activity mapping	[12]	Sensitivity: 92%, Specificity: 89%

**Table 3:** The comparison of deep learning models for radiology analysis: applications, techniques, advantages, and limitations.

Model	Application	Technique	Advantages	Limitations	Reference
YOLOv4	Tumor Detection in CT scans	Deep Learning (CNN-based)	High accuracy, fast real-time detection	Limited interpretability, struggles with small tumors	[15]
FasterR-CNN	Breast Cancer Detection in Mammography	Region Proposal Networks + CNN	High accuracy in bounding box prediction	Sensitive to noise, requires large datasets	[16]
RetinaNet	Detection of Lung Nodules in Chest X-rays	Focal Loss with CNN	Handles class imbalance well	Performance drops with poor-quality images	[17]
U-Net	Tumor Segmentation in Brain MRI	Convolutional Autoencoder (CNN)	High segmentation accuracy, pixel-level prediction	Overfitting on small datasets	[18]
EfficientDet	Detection of Cardiac Abnormalities	EfficientNet-based Architecture	Efficient with limited resources	Reduced performance with complex image conditions	[19]
Conditional (C-DCNN)	Brain Tumor Classification (MRI)	Conditional Deep CNN + GAN-generated synthetic data	Improved generalization with synthetic data, robust classification	Dependent on GAN quality, computational cost for training	[20]
LLM-based Vision Transformers	Multi-organ Detection in CT/MRI	Transformer Architecture	Integrates multiple object detection tasks	High computational cost, needs large datasets	[21]
Faster R-CNN + LSTM	Longitudinal Tracking of Tumor Progression in CT	CNN for Detection + LSTM for Sequence Modeling	Captures temporal changes in tumor progression	Complex, slow for real-time applications	[22]
DeepLabV3+	Detecting Brain Lesions in MRI scans	Atrous Convolution + CNN	High precision for lesion detection	Computationally expensive, requires fine-tuning	[23]
Ensemble CNNs	Colon Polyp Detection in Colonoscopy images	Multiple CNN Architectures	Improved performance through ensemble learning	Computationally intensive, requires careful architecture selection	[24]
Mask R-CNN	Liver Lesion Detection and Segmentation in CT images	Mask R-CNN + CNN	Accurate lesion segmentation and detection	Struggles with small lesions and low contrast images	[25]

**Table 4:** Data and annotation challenges in developing AI models for radiology.

Challenge	Summary	Key Findings	Reference
Data Preparation	Extensive time required for data cleaning, formatting, and image selection.	Efficient preprocessing pipelines and automated tools can reduce preparation time and improve dataset quality.	[26]
Annotator Expertise	Skilled annotators with expertise in annotation and healthcare are needed.	Training programs and collaboration with medical professionals can improve annotator expertise.	[27]
Labeling Consistency	Consistency in labeling is crucial for reliable model training.	Standardized annotation protocols and regular quality checks can enhance labeling consistency.	[28]
Limited Data Access	Limited access to diverse and representative medical images.	Data sharing agreements and federated learning approaches can improve data access while ensuring privacy.	[29]
Data Bias	Bias in medical datasets can lead to inaccurate model predictions.	Ensuring diverse datasets and using techniques like data augmentation and synthetic data can address bias.	[30]
Privacy Concerns	Patient privacy is a significant concern in medical image annotation.	Anonymization techniques and secure data handling are necessary to protect privacy.	[31]
Annotation Tools	The choice of annotation tools impacts efficiency and accuracy.	Feature-rich, user-friendly tools can improve annotation efficiency and reduce errors.	[32]
Quality Control	Regular quality checks are essential to maintain high annotation standards.	Multi-level review processes and automated quality control tools can enhance annotation reliability.	[33]
Scalability	Scaling annotation efforts to large datasets is challenging.	Crowdsourcing platforms and scalable annotation frameworks can help manage large-scale projects.	[34]
Integration with Clinical Workflows	Integrating annotation with clinical workflows enhances practical utility.	Collaboration with healthcare providers and tool integration into clinical systems can facilitate seamless data use.	[35]

**Table 5:** Overview of the Major gaps in the Application of Artificial Intelligence in Radiology.

Category	Subcategory	Issue	Example	Potential Solutions
Computational Challenges	Model Generalization	Variability in image quality, resolution, and acquisition parameters across modalities [36]	Models trained on high-resolution MRI performed poorly on low-resolution MRI [37]	Multimodal architectures; domain adaptation techniques [38]
	Resource Constraints	Real-time processing exceeds hardware capabilities in resource-constrained environments [39]	AI-powered ultrasound systems face delays in low-resource settings [40]	Model optimization through quantization and pruning [41]
Ethical and Legal Challenges	Bias and Fairness	Imbalanced datasets lead to disparities in diagnostic accuracy across populations [42]	AI for breast cancer detection underperformed for African-descent patient [43]	Diverse datasets; fairness-aware training strategies [44]
	Accountability and Liability	Undefined responsibility in AI-related errors [45]	AI stroke detection misclassification delayed treatment [46]	Comprehensive AI usage guidelines; standardized error analysis protocols [47]
Integration Challenges	Interoperability	Incompatibility of AI tools with health systems like PACS and EHR [48]	CT anomaly detection system failed to integrate with legacy PACS [49]	Standards adherence (DICOM, HL7 FHIR); collaboration with vendors
	Clinician Acceptance	Lack of trust due to AI’s black-box nature [50]	Skepticism about lung nodule detection AI due to limited explainability [51]	Focus on explainability, user-friendly interfaces [52]
Performance Challenges	Robustness to Variations	Inconsistent imaging conditions like motion artifacts and positioning impact performance [53]	High false positives in X-rays with motion artifacts [54]	Augmented datasets; preprocessing for normalization [55]
	Handling Rare Cases	Poor detection of rare conditions due to underrepresentation in training [56]	AI failed to identify rare glioblastoma subtype [57]	Synthetic data generation; transfer learning [58]
Economic Challenges	High Costs	High initial investments deter smaller facilities [59]	AI imaging system upgrade cost over USD 1 million for a tertiary hospital [60]	Public–private partnerships; subsidized solutions [61]
	Workforce Training	Training lack of AI familiarity hampers effective usage [62]	Radiologists needed six months to learn AI diagnostic tools [63]	Comprehensive training programs; AI in medical education [64]
